# Naxos Disease and Related Cardio-Cutaneous Syndromes

**DOI:** 10.1016/j.jacadv.2024.101547

**Published:** 2025-01-10

**Authors:** Alexandros Protonotarios, Angeliki Asimaki, Cristina Basso, Zafeirenia Xylouri, Emanuele Monda, Ioannis Protonotarios, Giulia Crisci, Dominic JR. Abrahms, Aris Anastasakis, Loizos Antoniades, Athanasios Bakalakos, Andreina Carbone, Aman S. Coonar, Juan Ramon Gimeno, George Lazaros, Stamatis Lerakis, Luisa Mestroni, George Papadopoulos, Leandro Pecchia, Francesca Romana Prandi, Petros Syrris, Julia Cadrin-Turigny, Anargyros Vasilakis, Jeffrey E. Saffitz, Stamatios Gaetano Thiene, Perry M. Elliott, Juan Pablo Kaski, William J. McKenna, Eduardo Bossone, Giuseppe Limongelli, Adalena Tsatsopoulou

**Affiliations:** a Centre for Heart Muscle Disease, UCL Institute of Cardiovascular Science, London, UK; b Cardiovascular and Genomics Research Institute of City, St George’s University of London, London, UK; c University of Padua, Medical School, Padua, Italy; d Acute Medicine, Southampton General Hospital, Southampton, UK; e Department of Traslational Sciences, University of Campania ‘Luigi Vanvitelli’, Naples, Italy; f University of Naples Federico II, Naples, Italy; g Department of Cardiology, San Paolo Hospital, University of Milan, Milan, Italy; h Center for Cardiovascular Genetics, Boston Children’s Hospital, Harvard Medical School, Boston, USA; i Inherited Cardiovascular Diseases, Onassis Cardiac Surgery Centre, Athens, Greece; j University of Cyprus Medical School, Nicosia, Cyprus; k Cardiothoracic Surgery, Royal Papworth Hospital, Cambridge University Health Partners, Cambridge, UK; l Inherited Cardiac Disease Department (CSUR/ ERN Guard Heart), Virgen de la Arrixaca University Hospital, Murcia, Spain; m George Lazaros, First Cardiology Department, School of Medicine, Hippokration General Hospital, National and Kapodistrian University of Athens, Athens, Greece; n Department of Cardiology, Mount Sinai Fuster Heart Hospital, Icahn School of Medicine at Mount Sinai, New York, NY; o Medicine/Cardiology, Genetics Program, University of Colorado Cardiovascular Institute, Aurora, USA; p University of Athens Medical School, Athens, Greece; q University Campus Biomedico, Rome, Italy; r Weill Cornell, NY Presbyterian Hospital, New York, USA; s Cardiovascular Genetics Center, Montréal Heart Institute, Faculty of Medicine, University of Montréal, Montréal, Québec, Canada; t Representative of Naxos disease patients, Naxos, Cyclades, Greece; u Harvard Medical School, Department of Pathology, Beth Israel Deaconess Medical Center, Boston, USA; v Precision Medicine Network in Cardiology Onassis Cardiac Surgery Center, Athens, Greece; w Pediatric Clinic, Naxos, Cyclades, Greece

**Keywords:** Naxos disease, cardio-cutaneous syndrome, arrhythmogenic right ventricular cardiomyopathy, arrhythmogenic cardiomyopathies, plakoglobin, desmoplakin

## Abstract

Naxos disease is a rare autosomal recessive condition combining arrhythmogenic right ventricular cardiomyopathy, woolly hair, and palmoplantar keratoderma. The first identified causative variant was in the gene encoding the desmosomal protein plakoglobin. Naxos disease exhibits fibro-fatty myocardial replacement with immunohistological abnormalities in cardiac protein and signaling pathways, highlighting the role of inflammation and potential anti-inflammatory treatments. Childhood cutaneous signs precede cardiac features, which are diagnosed by familial and genetic evaluation, electrocardiography and cardiac imaging. Disease progression necessitates holistic care with risk management and lifestyle adjustments, often needing treatment for arrhythmia and heart failure. Similar phenotypes have been linked to desmoplakin and rarely desmocollin2 gene variants, highlighting the importance of familial and genetic evaluation. This document summarizes current knowledge on Naxos disease and related cardiocutaneous syndromes and initiates an international endeavor to collect and study all global cases, aiming to improve understanding, treatment, and patient care through shared data and research.

## Highlights

• The cutaneous phenotype of Naxos disease (palmoplantar keratoderma, with or without skin erosions or bullous lesions, combined with hair shaft changes—woolly hair or occasionally absence of hair) is a red flag for thorough cardiac screening for arrhythmogenic cardiomyopathy at any age.
• The diagnosis of Naxos disease or any other cardiocutaneous syndrome warrants familial and genetic evaluation.
• Symptoms including palpitation, chest pain, and/or syncope typically present during adolescence.
• The earliest disease manifestations are the cutaneous features and electrocardiographic abnormalities which typically precede the development of symptoms and/or arrhythmia.
• Disease progression with arrhythmia and heart failure warrants comprehensive, tailored treatment and lifestyle modifications.
• Global collaboration aims to better understand cardiocutaneous disease to enable development of targeted treatments to enhance patient longevity and quality of life.

### Definition and the aim of an international endeavor

**Figure 1:**
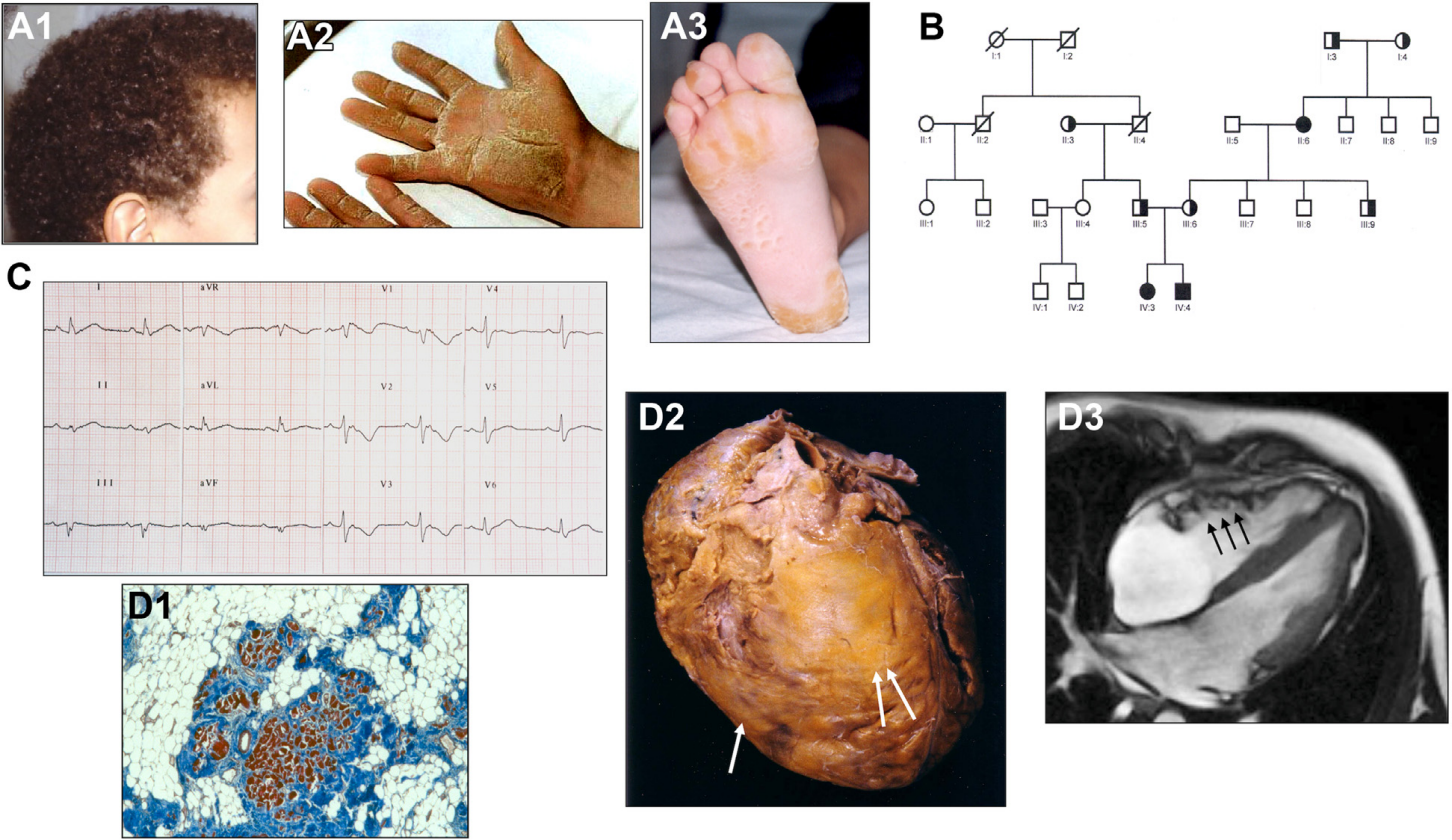
**Summary Features in Naxos Disease Patients (*JUP2157del2*)** (A) Characteristic cutaneous features in a patient with Naxos disease: 1. Woolly hair, 2. Palmar and 3. Plantar keratoderma. (B) Pedigree of a family with Naxos disease (*JUP 2157*), showing the autosomal recessive mode of patient distribution. Solid shapes: Homozygous carriers with 100% penetrance of disease. Half-marked shapes: Heterozygous carriers without clinically detectable heart and skin phenotype. (C) 12-lead ECG from an adult patient with Naxos disease (*JUP 2157*): QRS prolongation, epsilon waves in V1-V3, T-wave inversion in V1-V4 and low voltage. (D) Structural myocardial changes, detected in patients with Naxos disease (*JUP 2157*). 1: Myocardial histology (Masson’s trichrome stain) revealing degenerating myocardial fibres (red) surrounded by fibrous (blue) and fatty (white) tissue; 2: Postmortem sample of a whole heart, anterior view, revealing a dilated RV (inflow, apex and outflow) (2 arrows) and a small part of the dilated LV (one arrow). 3: Kinetic abnormalities appearing as “accordion sign” (arrows) on the RV-free wall as shown on CMR. CMR = cardiac magnetic resonance; ECG = electrocardiography; LV = left ventricular; JUP = junctional plakoglobin; RV = right ventricular.

Naxos disease is a rare, autosomal recessive cardiocutaneous disorder characterized by arrhythmogenic right ventricular cardiomyopathy (ARVC), woolly hair (WH), and palmoplantar keratoderma (PPK) (Figure 1). It was first described by Nikos Protonotarios et al in 1986, on 9 patients across 4 families, originating from the Greek Naxos Island.^1^

The discovery of a junctional plakoglobin (*JUP*) variant in Naxos disease highlighted the role of desmosomal proteins in cardiomyopathies.^2^ Research has shown that desmosomal gene variants can cause both isolated cardiac and syndromic forms of ARVC and may overlap with dilated cardiomyopathy (DCM).^3^, ^4^, ^5^ In the following years, population screening in the Aegean islands showed that the disease occurs in about 1 in 1,000 people in certain regions.^6^ The discovery of the same JUP variant in a family from eastern Turkey suggested a historical connection between these populations. Other desmosomal gene variants in *JUP*, desmoplakin (*DSP*), and desmocollin 2 (*DSC2*) have been found in isolated cases of similar cardio-cutaneous syndromes in various parts of the world, from the Mediterranean to Argentina and Ecuador. However, no systematic study has been conducted to determine the overall prevalence of these syndromes.

This document aims to summarize current knowledge on Naxos disease and similar syndromes, advocating for a global effort to collect and study cases.

## First part: Disease mechanisms

### Genetics

The concurrence of hair and skin features with ARVC in 21 affected individuals across 9 Naxos families (from a cohort of 150 individuals) provided a clear diagnostic marker, effectively distinguishing those with the cardiomyopathy. Meanwhile, pedigree analysis suggested autosomal recessive inheritance. These led to homozygosity mapping and linkage analysis, which pinpointed the disease locus to 17q21.^7^ Subsequently, the recessive *JUP c.2157del2* variant was identified.^2^ Shortly after, the recessive *DSP 7901delG* variant was discovered in 6 patients from 3 Ecuadorian families, underlying a phenotype of PPK and WH similar to Naxos disease, but with a cardiomyopathy phenotype more closely resembling DCM.^4^

Other variants in *JUP, DSP*, and *DCS2* were later found to cause similar cardiocutaneous disorders with various inheritance patterns. All cases exhibited both cardiac and skin features (Supplemental Table). In a study involving 6 families with dominant loss of function *DSP* variants, the presence of a phenotype of curly hair and minor PPK features cosegregated with arrhythmogenic cardiomyopathy phenotype.^8^

There is only 1 report of 2 siblings with ARVC and left ventricular (LV) involvement, associated with cutaneous features of mild PPK and WH, related to a homozygous single-base deletion in *DSC2 (1841delG*)^9^ (Supplemental Table).

## Epidemiology: Worldwide geographical map

Since the initial description of Naxos disease, additional cardiocutaneous cases have been documented worldwide.

Besides Naxos Island, affected families with the same variant in *JUP* were found on other Aegean islands. The disease prevalence in some Aegean regions was estimated at 1:1000.^6^ Additionally, the same variant with a similar cardiac and skin phenotype has been reported in Turkey.^10^

Other homozygous *JUP* variants, linking arrhythmogenic cardiomyopathy with WH, alopecia, or PPK, have been reported from Argentina and Canada and have been additionally associated with lethal neonatal epidermolysis, total alopecia, and onycholysis (Supplemental Table).

A decade after Naxos disease was described in 1986, a similar condition involving DCM with early arrhythmias was described in Ecuadorian families.^11^ This was linked to a *DSP* variant and named Carvajal syndrome.^12^^,^^13^

Other *DSP* variants underlying similar to Naxos disease cutaneous features with a closer to DCM phenotype were subsequently reported worldwide. One family with a recessive variant in *DSC2* inherited in homozygosis has been reported from the United Kingdom.^9^

**Figure 2:**
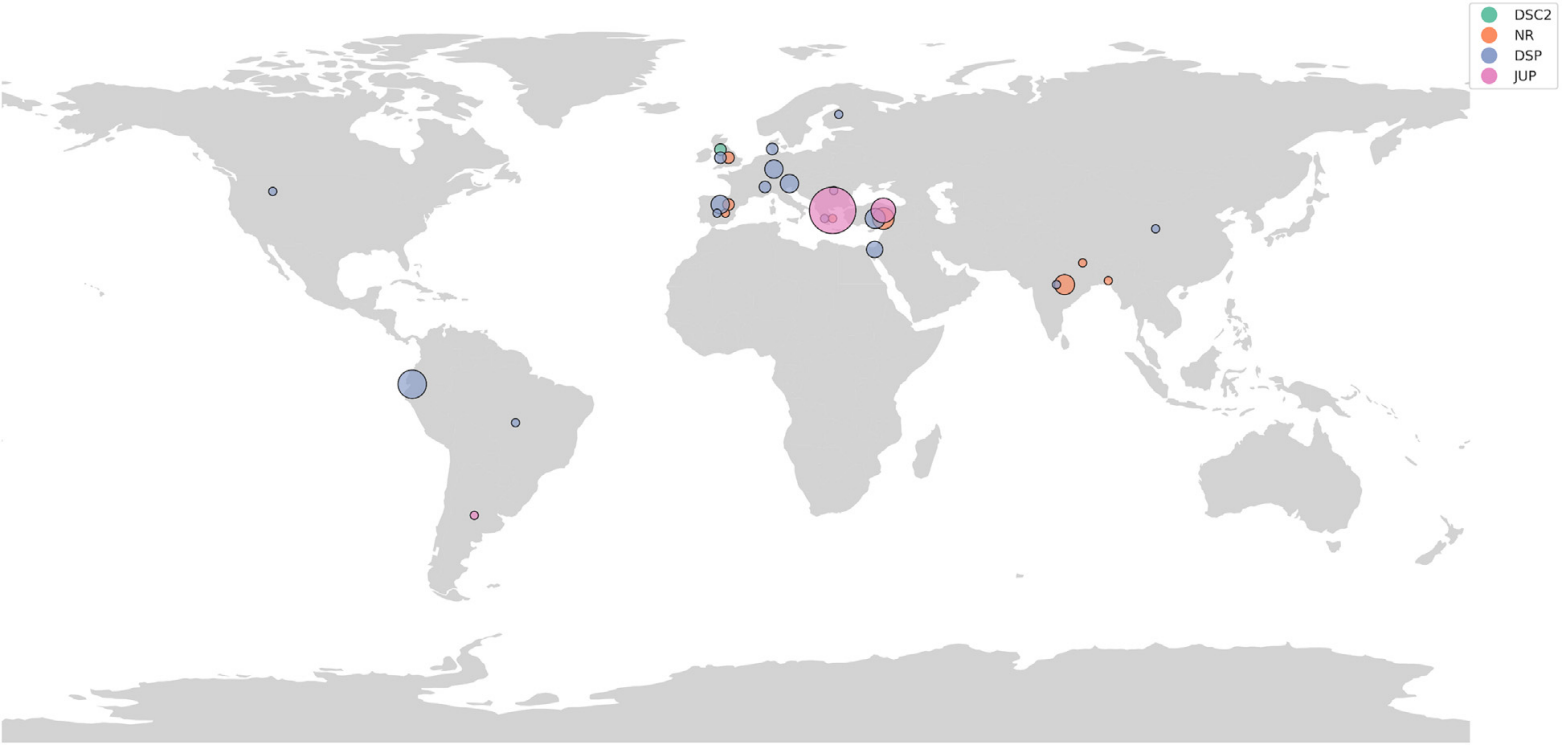
**Map of Global Distribution of Naxos Disease and Related Cardio-Cutaneous Cases, as Reported up to 2024** Global distribution of Naxos disease and related cardio-cutaneous cases. Each circle represents the presence of reported cases within a country, color-coded by the gene involved. Circle size is proportional to the number of cases reported, with larger circles indicating a higher number of cases. Jitter is added to minimize overlap. This map provides a visual summary of the global epidemiology of Naxos-like syndrome, highlighting the geographical distribution and prevalence of genetic variants associated with the condition.

The global distribution and number of reported cases are detailed in the Supplemental Table, with a visual representation provided in Figure 2.

### Pathology and disease mechanisms

#### Cardiac pathology-histopathology

**Figure 3:**
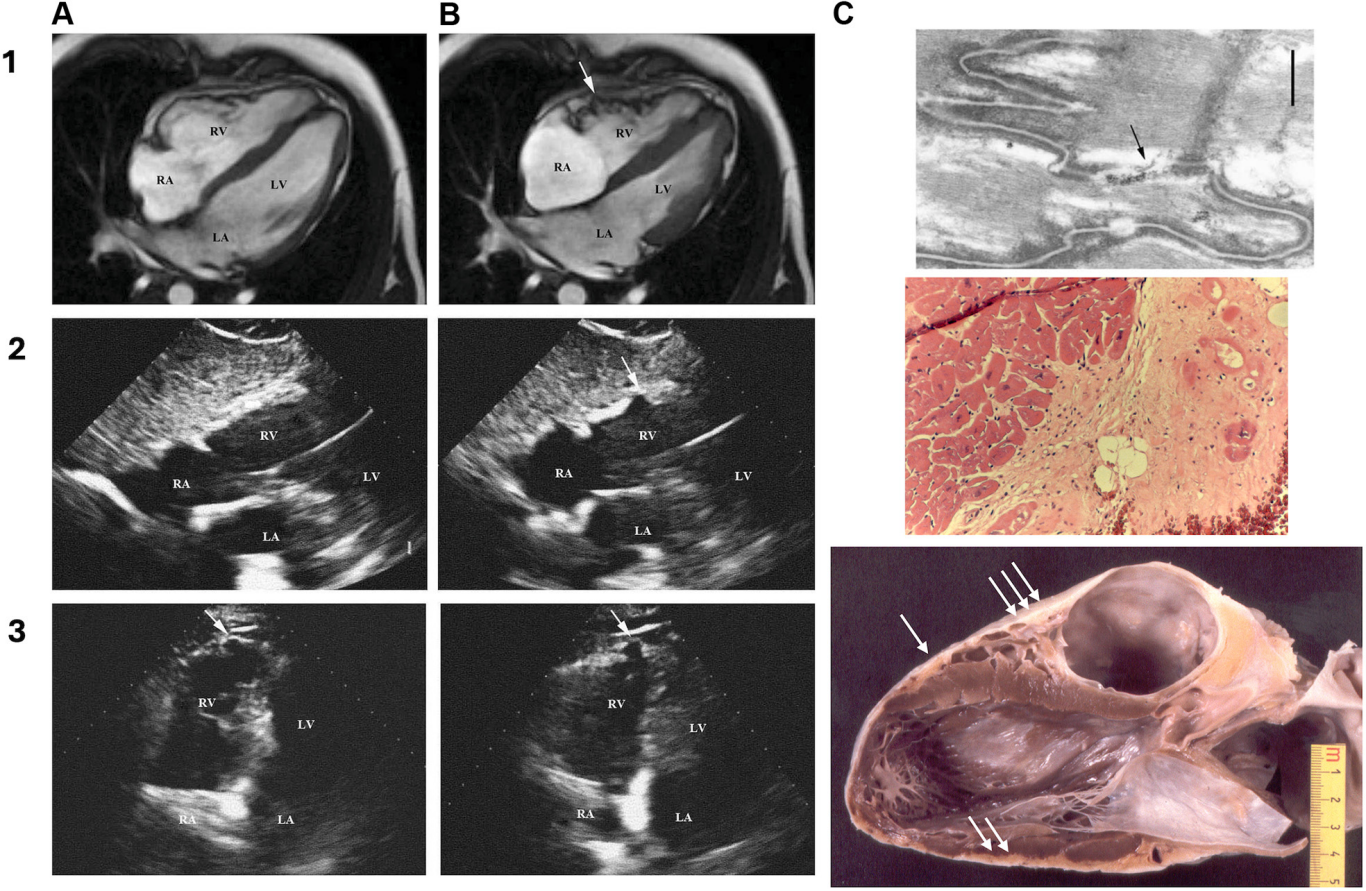
**Functional and Structural Myocardial Changes in Patients with Naxos Disease (*JUP2157del2*)** (A) Diastole and (B) systole. On CMR: 1, and 2D-ECHO: 2 and 3. Arrows pointing on RV dyskinetic areas. RV free wall dyssynchronous contraction (accordion sign): 1B, RV posterior wall dyskinesia with systolic bulging (retraction sign): 2B, and apical RV aneurysm: 3A and 3B (akinesia/dyskinesia with diastolic bulging) C1: An electron micrograph shows a decreased gap junction profile length in the LV myocardium (Bar 0.5 μm). C2: Postmortem histology (haematoxylin and eosin stain; cell nuceli: blue, cytoplasm: pink, extracellular fibrosis: light pink,) from LV myocardium, showing myocyte loss with predominantly fibrous replacement of myocardial tissue. C3: Gross examination on postmortem (the patient died of heart failure waiting for heart transplantation), showing on the parasternal long-axis view of the heart, paper-thin RV walls, transmural myocardial loss (one arrow), subepicardial left ventricular fatty infiltration (two arrows), and calcified fibrous plaque at the level of the RV outflow tract (three arrows). ECHO = echocardiography; other abbreviations as in Figure 1.

Naxos disease patients show classic features of fibro-fatty replacement of atrophic myocardium, extending from the epicardium to the endocardium, typical of autosomal dominant ARVC (Figures 1, D1 and 3, 3C).^14^ In 4 Naxos disease patients, 2 had typical ARVC with biventricular involvement, 1 had predominant right ventricular (RV) and focal LV involvement, and 1 had no histopathological changes, dying of leukemia before phenotypic expression at age 7 suggesting that the Naxos cardiac phenotype is acquired with age, similar to nonsyndromic ARVC. One patient exhibited extensive calcification of the RV outflow tract, possibly explaining decreased arrhythmogenicity before dying from heart failure (Figure 3, C3).^15^

Carvajal syndrome, a cardiocutaneous syndrome related to Naxos disease, presents with cutaneous features that are clinically indistinguishable from those of Naxos disease, along with a cardiac phenotype resembling DCM, often accompanied by early ventricular arrhythmias and heart failure.^13^ A whole heart from a patient with Carvajal syndrome was examined postmortem. The cardiac pathology features eccentric ventricular hypertrophy and focal aneurysms in the RV, without fatty replacement, but with fibrous tissue, often seen in autosomal dominant *DSP* disease with a predominant LV phenotype.^16^

#### Cardiac and extracardiac immunohistology

**Figure 4:**
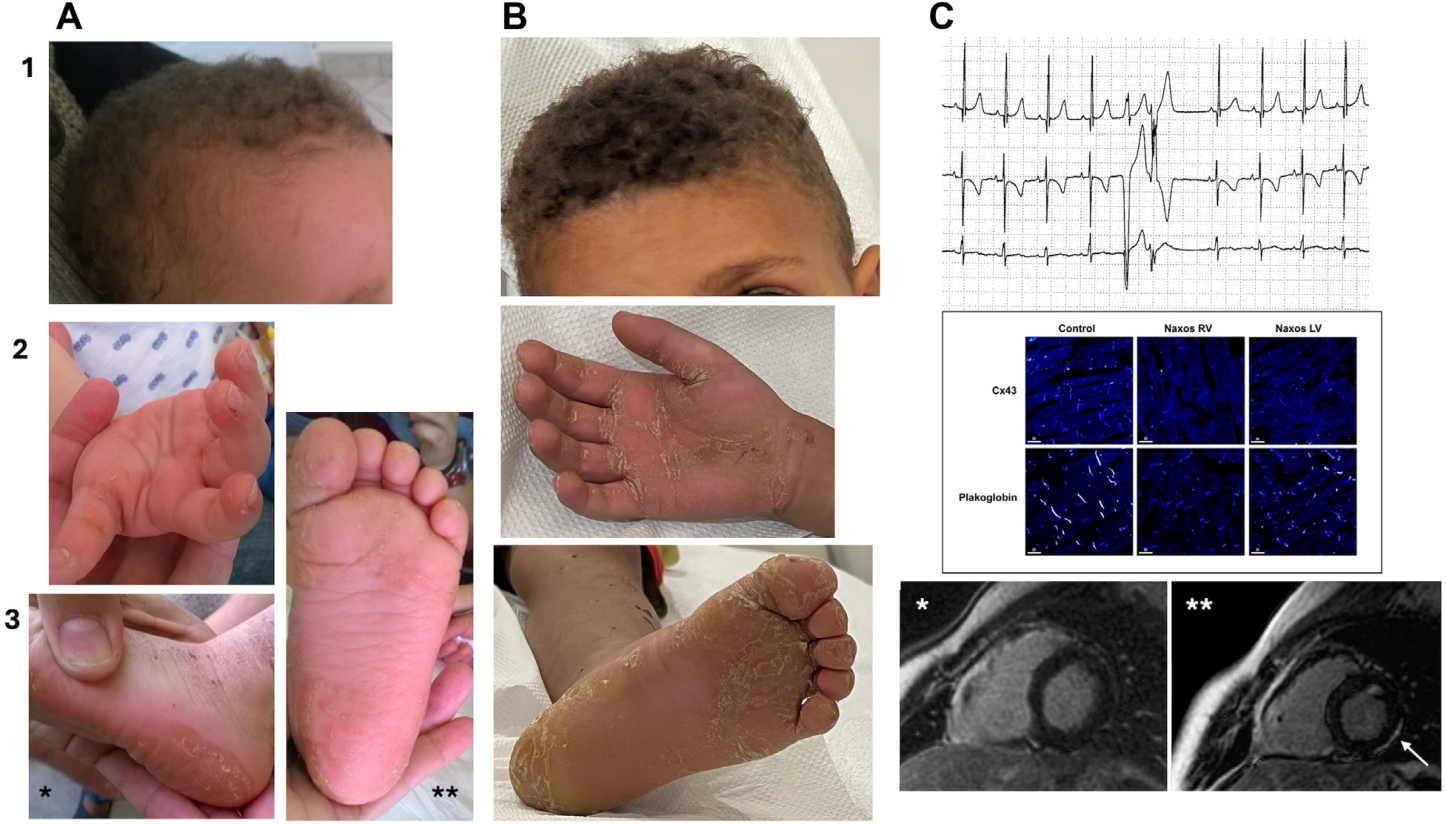
**Naxos Disease, Early Phenotype in *JUP* 2157del2 Homozygous Children** Cutaneous features can be detected since early childhood: Woolly hair at 14 months (1A) and 7 Years (1B). PPK mild at 14 months (2A, 3A∗∗) is potentially associated with eczematous lesions on the external foot and leg surfaces that usually resolve with age (3A∗). PPK shows a striate appearance at the age of 7 years 2B, 3B. Cardiac phenotype usually presents with an arrhythmic profile. 1C: Ventricular ectopics (14,000/day) detected on an asymptomatic child screened because of woolly hair and PPK at the age of 4 years, while she presented a normal for her age 12-lead ECG and normal 2D-ECHO. 2C: Abnormal distribution of connexin43 and plakoglobin on the right and left ventricular myocardium, detected on postmortem, after a non-cardiac death, while 2D-ECHO, gross pathology and regular histology were normal.^52^ ARVC in Naxos disease may present as an episode of acute myocarditis. 3C: Late contrast-enhanced image in a 14-year-old boy with Naxos disease, homozygous for *JUP 2157*, showing LGE (arrow) in LV (3C∗∗) as compared to (3C∗) image taken taken one year prior to (3C∗∗). The boy, followed-up yearly since the age of one year due to cutaneous features of WH and PPK, was asymptomatic till the age of 14 years, when he first presented with chest pain and troponin elevation. PPK = palmoplantar keratoderma; other abbreviations as in Figures 1 and 3.

Naxos disease patients were among the first ARVC patients whose myocardial samples were subjected to immunohistochemistry analyses, revealing that mutant JUP is expressed (as evidenced by Western immunoblotting) but fails to localize at the cardiac intercalated disks (IDs) (Figure 4, 2C).^17^ Immunoreactive signal for JUP is significantly depressed at the IDs in all forms of ARVC regardless of the underlying mutation.^18^ Furthermore, it has been shown that there is gap junction remodeling in Naxos disease (Figure 3, 1C).^17^ Loss of immunoreactive signal for Cx43 was also evident in myocardial samples from a 7-year-old girl homozygous for the *JUP2157del2* variant who died of leukemia prior to developing any histological or functional cardiac abnormalities (Figure 4, 2C).^17^ These findings support the hypothesis that gap junction remodeling appears to precede structural alterations being key contributor to arrhythmogenesis.

Immunohistology on postmortem examination of an Ecuadorian patient's heart revealed diminished signals for *DSP*, plakoglobin, and connexin43 at intercellular junctions, suggesting compromised desmosomal and gap junction integrity. Additionally, desmin was abnormally localized, failing to appear at the IDs.^13^

Studies have shown that buccal epithelial cells express all aforementioned protein markers and may thus act as a surrogate for the heart.^19^ Immunoreactive signal for JUP was lost in buccal cells from 6 patients with Naxos disease while signal for connexin43 (Cx43) was severely depressed in 5/6. These findings suggest that buccal mucosa immunohistology may have significant potential as an easy-to-acquire disease biomarker.

#### Intracellular pathways

JUP contributes to both adhesive and signaling functionalities, as it is involved in the Wnt signaling pathway.^20^^,^^21^ Desmosomal perturbations can lead to nuclear accumulation of plakoglobin and downregulation of the canonical Wnt signaling and thereby enhancement of adipogenesis driven by peroxisome proliferator-activated receptor γ and CCAAT-enhancer-binding protein alpha.^22^^,^^23^ Furthermore, ARVC-causing mutations lead to dislocation of protein kinase C alpha and Wnt inhibition and enhanced adipogenesis via the Hippo pathway.^24^ GSK3β is also implicated as its pharmacological inhibition prevents and reverses the ARVC phenotype (including arrhythmias, contractile dysfunction, myocyte injury, apoptosis, exercise-induced sudden cardiac death (SCD), and redistribution of junctional proteins) in ARVC models in vitro, in vivo, and ex vivo including transgenic zebrafish and mouse models with cardiac-specific expression of the *JUP2157del2* variant.^23^^,^^25^ Modeling of aberrations in other desmosomal proteins support the involvement of key components in Ca^+2^ signaling pathways that might be part of a final common pathway in all forms of ARVC.^26^^,^^27^

#### Inflammation

Myocardial infiltrates are observed in >70% of ARVC myocardial samples, predominantly in areas with extensive fibrofatty replacement and contain T-lymphocytes, macrophages, neutrophils, and mast cells.^14^^,^^28^ Myocarditis-like “hot-phases” have been reported in Naxos disease patients, who present with ST-segment elevation and/or increased troponin levels.^29^^,^^30^ Several experimental models have been used to gain insights into the role of inflammation in the pathogenesis of ARVC.

Neonatal rat ventricular myocytes transfected to express the *JUP2157del2* variant express and release high levels of pro-inflammatory mediators in their culture medium including tumor necrosis factor-alpha, interleukin-6, macrophage inflammatory protein-1 alpha, and regulated upon activation normal T-cell expressed and secreted.^23^ NFκB is a master regulator of inflammation and its inhibition in disease models reduced inflammatory cytokine production and prevented key protein redistribution and apoptosis.^31^ Understanding the precise role inflammation plays in disease progression could aid diagnosis and risk stratification as well as provide the basis for the development of mechanism-based therapies targeting aberrant immune activation.^32^

### Novel therapeutics

Mechanistic insights from animal and cell models that aim to replicate the cardiac features of Naxos disease have the potential to uncover key molecules that can dampen disease progression, such as by modulating the innate inflammatory response.^31^ The most promising therapeutic pipeline, however, remains that of gene therapy, with numerous trials currently ongoing for genetic cardiomyopathies, especially where the mechanism is underlied by gene haploinsufficiency.^33^ Specialized funding programs and industry partnerships are crucial for developing and increasing access to novel therapeutics for Naxos disease, given its rarity and the limited viability for extensive pharmaceutical research.

## Second Part: The Patient Pathway

### Diagnostic workup

#### Clinical presentation and natural history: Transition from pediatric to adult care

Naxos disease's cutaneous features are evident from infancy, characterized by woolly, dense hair, curly eyebrows, and eyelashes (Figure 4). PPK with occasional hand and legs eczematous lesions appears within the first 2 years (Figure 4). Hyperkeratotic plaques have a distinct erythematous border. Some *JUP* variants may show absent or sparse hair (Supplemental Table).

About half of Naxos disease patients have syncopal episodes or develop episodes of sustained ventricular tachycardia, between their teenage and 35 years. In the series studied by Protonotarios N et al, half of patients experienced arrhythmic events during the disease course.^34^ Ventricular tachycardia on electrocardiography (ECG) consistently showed a left bundle branch block pattern.^35^ Atrial fibrillation was documented in 3 cases.^1^^,^^36^ A 5-year-old girl with the *JUP-2157del2* variant exhibited frequent RV extrasystoles on her ECG despite a normal echocardiogram (Figure 4, 1C). Following a noncardiac death, postmortem histopathology showed gap junction remodeling without fibrofatty replacement.^17^ The disease generally progresses with RV function deterioration, new wall motion abnormalities, and potential LV function decline.^37^ Episodes of arrhythmic storms “hot phases” may trigger structural deterioration or sudden death, sometimes resembling acute myocarditis in children or adolescents (Figure 4, 3C).^29^^,^^38^^,^^39^ Comprehensive cardiac evaluation is crucial for children or adults presenting with chest pain to detect ARVC progression.

In Ecuadorian families, with Carvajal syndrome, all affected members showed electrocardiographic and/or echocardiographic abnormalities, with the youngest cases appearing by age 7. Resting 12-lead ECGs were abnormal in all, commonly showing low voltage and intraventricular conduction defects.^11^ T-wave inversion in V_1_-V_3_, sometimes extending to V_5_, was frequently observed.^40^ All patients who underwent 24-hour Holter monitoring showed complex ventricular extrasystoles and episodes of nonsustained ventricular tachycardia. Over 90% developed LV involvement by their second decade, often severe. Though the right ventricle was also affected, detailed evaluation was limited.^40^ Fifty-seven percent of patients developed heart failure, with most dying during adolescence, likely from heart failure, though arrhythmic death cannot be excluded due to limited out-of-hospital data.^4^ The pronounced arrhythmogenicity and ECG abnormalities in these patients are now recognized as part of the nondilated LV cardiomyopathy phenotype spectrum.^5^

Heart failure symptoms, though rare at onset, can develop and may necessitate heart transplantation in some cases,^6^ particularly in those with *DSP* variants (Supplemental Table). In the largest longitudinal cohort with Naxos disease, annual disease-related mortality was 3%, with sudden death mortality at 2.3%.^37^ Sporadic reports of homozygous *JUP* variants include a toddler's sudden death, an 18-year-old's heart transplantation,^41^ and 2 cases of congenital epidermolysis bullosa and infantile death, 1 with confirmed cardiac involvement (Supplemental Table).

#### Electrocardiography

**Figure 5:**
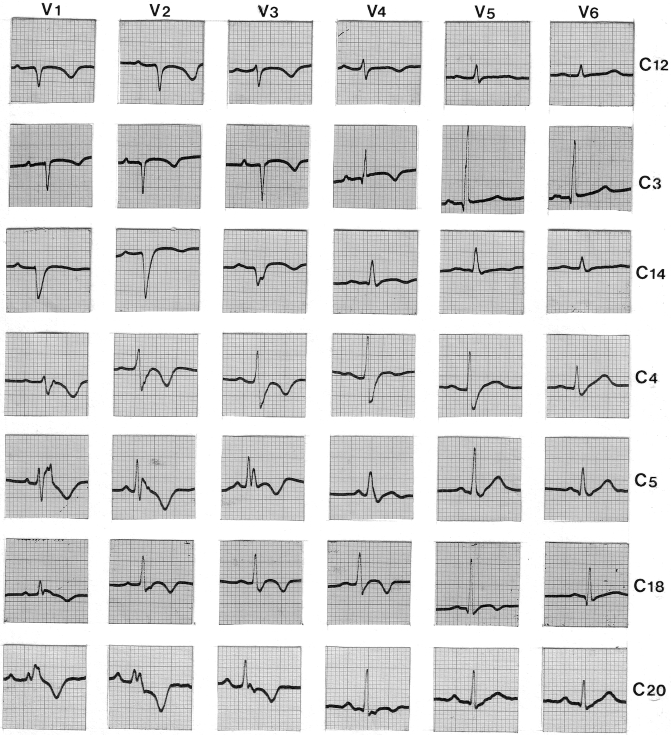
**Electrocardiography Spectrum in Naxos Disease Patients** Samples from 12-lead ECG precordial recordings in seven different patients with Naxos disease (*JUP 2157del2*): A broad spectrum of QRS width, amplitude and morphology, as well as the distribution of T-wave inversions, is noted. C-denotes the case number. Abbreviations as in Figure 1.

ECG abnormalities may appear early, preceding structural changes as shown in a boy with Naxos disease homozygous for *JUP* 2157del2 variant who was first investigated the age of 6 years due to the cutaneous features and then followed up regularly (Table 1). ECG changes might indicate right or left ventricle involvement.^42^^,^^43^ In the largest Naxos disease series, over 90% of patients showed a broad spectrum of ECG abnormalities as symptoms emerged (Figure 5). Electrocardiographic abnormalities included inverted T waves in leads V_1_ to V_3_ or across the precordial leads, epsilon waves, QRS complex prolongation in V_1_ to V_3_, and right bundle branch block (complete or incomplete) (Figure 5).^37^ Adolescents may experience T-wave reinversion in V_2_ and V_3_. Epsilon waves, present in 34% of patients, were associated with right ventricular outflow tract wall motion abnormalities and increased RV diameter.^44^ Low voltage and flat T waves in left precordial leads were seen in severe right and/or biventricular disease, with frequent ventricular extrasystoles and/or ventricular tachycardia in over 90% of cases.^37^ Ventricular extrasystoles of right bundle branch block configuration were less common.^34^^,^^37^

**Table 1:** Early Prestructural, ECG Evolution in a Boy With Naxos Disease

	Age, y										
	6	7	8	9	10	11	12	12.5	13	14	15
TWI	V1	V1	V1	V1	V1	V1	V1	V1	V1	V1	V1
TAD (V1)	36	36	44	56	51	**58**	**60**	**60**	**64**	**64**	**60**
QRSf (ms)	104	102	107	115	111	**123**	**125**	**127**	**127**	**141**	
LAS (ms)	32	31	33	37	35	**48**	**42**	**44**	**54**	**60**	
RMS (μV)	46	62	44	20	31	**11**	**11**	**9**	**7**	**9**	
VES/24h	0	0	0	1	2	66	374	**1684**	**504**	**508**	**1814**
ECHO	N	N	N	N	N	N	N	**N**	**N**	**N**	**N**
CMR	-	-	-	-	-	-	-	**-**	**N**	**N**	**-**

ECHO = echocardiography; CMR = cardiac magnetic resonance; LAS = low-amplitude signal duration; QRSf = filtered QRS duration; N = within normal limits; TAD = terminal activation duration; TWI = T-wave inversions; RMS = root-mean-square voltage of terminal 40 ms; VES = ventricular extrasystoles; ECG = electrocardiography.

Patients with DSP cardiocutaneous disease and a DCM phenotype often exhibited T-wave inversion in inferolateral leads, low QRS voltages, and abnormal Q waves (Supplemental Table).^4^^,^^42^^,^^45^, ^46^, ^47^ Resting 12-lead ECG and 24-hour Holter monitoring are essential in diagnosing cardiocutaneous phenotype, as electrical abnormalities are early signs of cardiomyopathy with a high incidence of ventricular arrhythmias.^4^

#### Cardiac imaging

Typical transthoracic echocardiography findings in Naxos disease resemble dominant ARVC (Figure 1), showing RV outflow tract dilation, dysfunction, and focal dyskinetic areas, often involving the RV apex and mid/apical lateral walls, with aneurysmal dilation also described.^37^^,^^45^^,^^48^, ^49^, ^50^, ^51^ In the largest Naxos disease series, RV alterations ranged from mild dilatation or regional hypokinesia (27%) to severe dilatation, diffuse hypokinesia, and aneurysms (73%), with LV abnormalities in 25% of patients.^34^^,^^37^^,^^51^

DSP disease typically presents with a DCM phenotype, featuring LV and/or biventricular dilation and impaired systolic function, occasionally with LV intracavitary thrombus formation (Supplemental Table).^4^^,^^42^^,^^45^, ^46^, ^47^

Cardiac magnetic resonance (CMR), the gold standard for noninvasive RV assessment, offers excellent spatial resolution, 3 dimensional imaging, high reproducibility, and prognostic value, with right ventricular ejection fraction <40% predicting major cardiac events in CVD patients.^52^ While less common in Naxos disease reports, CMR findings align with echocardiography, and late gadolinium enhancement can assess myocardial fibrosis (Figure 3).^15^^,^^29^^,^^53^ CMR aids arrhythmic risk stratification in ARVC, potentially guiding intracardiac cardioverter defibrillator (ICD) implantation decisions, as right ventricular ejection fraction by CMR predicts appropriate ICD shock or death in ARVC patients.^54^ Although cardiac CT data in Naxos disease are unavailable, CT can differentiate ARVC from other tachyarrhythmias and guide ventricular tachycardia ablation and risk stratification despite its exclusion from the 2010 ARVC task force criteria.^55^^,^^56^

#### Recommendations for screening family members: genetic testing and genetic counseling

Naxos disease and related cardiocutaneous syndromes are inherited mostly in an autosomal recessive pattern.^3^ The most extensively studied series of *JUP* variants exhibit full penetrance by adolescence, with variable ARVC severity.^37^ In addition, early cardiac features have been observed in cases with homozygous DSP variants (Supplemental Table). Occasionally dominant *DSP* variants associated with a cardiomyopathy phenotype exhibit minor cutaneous abnormalities.^8^ Therefore, in patients with WH and palmoplantar keratosis early genotyping and cardiac assessments (ECG, echocardiogram, Holter/ECG monitoring) is mandatory. In children with normal initial cardiac investigations CMR should be performed when this is feasible without general anesthesia or sedation, usually from 8 to 10 years of age. Regular evaluations every 1 to 2 years are advised (Central Illustration).

Genetic testing and counseling for children should prioritize their best interests. A confirmed pathogenic variant warrants family member screening for carrier status. Pretest and posttest counseling, crucial to the process, addresses cultural and legal factors, particularly in prenatal and preimplantation diagnostics, to support informed decisions in family planning and disease management. A likely pathogenic variant should be reassessed approximately yearly to determine if the designation has changed.^5^ Genetic counseling is essential for understanding inheritance risks, providing psychosocial support, and aiding informed decision-making in family planning and disease management (Central Illustration).^5^

**Central Illustration:**
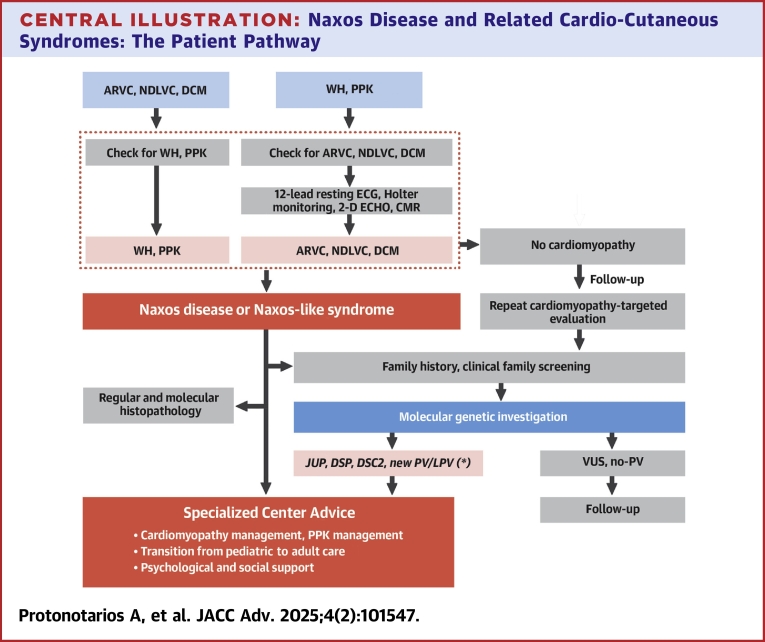
**Naxos Disease and Related Cardio-Cutaneous Syndromes: The Patient Pathway** The Patient Pathway is an opinion statement. ∗Any new P/LP variant identified either on the already involved genes of *JUP, DSP, DSC2* or in other genes. DSC2 = desmocollin 2; DSP = desmoplakin; other abbreviation as in Figure 1.

#### Endomyocardial biopsy

Though endomyocardial biopsy (EMB) has been a major criterion for diagnosing ARVC, since 1994,^55^ the use of CMR for tissue characterization increased recently, leading to a decline in EMB use, which is now primarily reserved for inconclusive noninvasive assessments or to exclude phenocopies like sarcoidosis and myocarditis, especially in sporadic cases.^57^ The "hot phase" of ARVC can complicate differential diagnosis with myocarditis (Figure 4, 3C). EMB is not without risks and should be performed by experienced cardiomyopathy teams, with electroanatomic voltage-guided EMB enhancing diagnostic accuracy.^58^^,^^59^

#### Multidisciplinary team, education, network organization, and artificial intelligence in managing Naxos disease and related syndromes

Managing Naxos disease and related cardiocutaneous syndromes requires a comprehensive, multidisciplinary approach aligned with cardiomyopathy guidelines.^5^ A multidisciplinary team (MDT) is essential, including pediatric and adult cardiologists, dermatologists, geneticists, genetic counselors, pediatricians, and pathologists to provide holistic care. The MDT ensures accurate diagnosis, family screening, and ongoing management. Educating patients and health care professionals is key to raising awareness and improving understanding of these rare syndromes.

Currently, no established networks exist for Naxos disease and related syndromes. Creating a network tailored to patient needs is crucial to overcoming diagnostic delays and coordinating care across primary care, specialized centers, and support services. This network should excel in diagnostics, therapy, and patient support, while considering geographic and socioeconomic diversity.

Artificial intelligence (AI) offers new opportunities in managing Naxos disease by improving screening, referral, and diagnosis. AI can enhance early identification of at-risk individuals, streamline referrals to specialized centers, and support personalized management in clinical settings. The successful AI integration will rely on collaboration between clinical experts, engineers, and AI specialists to harness its full potential in advancing patient care.

## Management

### Management of cardiac disease

#### Arrhythmias

The role of anti-arrhythmics in Naxos disease and similar cardio-cutaneous syndromes has not been specifically documented, so guidance from ARVC management is typically applied.^5^ Medical therapy for patients with ventricular arrhythmias aims to control symptoms, reduce arrhythmia burden, and mitigate ICD shocks. Beta-blockers are commonly used in ARVC for their safety, often as the first-line therapy, sometimes combined with flecainide. Amiodarone is a second-line therapy with variable efficacy due to small sample sizes and differing disease stages among patients.^60^, ^61^, ^62^ Its use in children with Naxos disease requires careful consideration of potential side effects.

According to the ESC guidelines for cardiomyopathies (Class IIa, Level of Evidence: C), catheter ablation, including the epicardial approach guided by 3D electroanatomical mapping, should be considered for patients with incessant ventricular tachycardia or frequent ICD interventions despite pharmacological treatment.^5^ Atrial fibrillation, which has been observed in the largest series of Naxos disease cases,^37^ should be managed according to the latest guidelines for cardiomyopathies.^5^

#### Heart failure

Heart failure management in ARVC involves treating LV and RV dysfunction. For LV dysfunction, the treatment in adults should align with recommendations from current international guidelines ^63^^,^^64^ and include beta-blockers, aldosterone antagonists, sodium-glucose cotransporter 2 inhibitors, and angiotensin receptor/neprilysin inhibitor. Conversely, in children, data on sodium-glucose transport protein 2 inhibitors are scant, and recent findings from the PANORAMA-HF trial seem to question the clinical superiority of angiotensin receptor/neprilysin inhibitor over angiotensin converting enzyme inhibitors.^65^^,^^66^ Treatment for RV dysfunction lacks specific trials, but common practice in experienced centers includes angiotensin converting enzyme inhibitors/angiotensin II receptor blockers, beta-blockers, and diuretics.

#### Risk stratification

Risk prediction and stratification for SCD are critical in managing patients, especially for identifying those needing ICD implantation. The first gene-specific risk study in ARVC focused on Naxos disease patients,^37^ revealing a high incidence of arrhythmia-related death and major events comparable to pre-ICD era ARVC literature. Despite nearly 100% penetrance of the cardiac component in Naxos disease, studies indicate similar arrhythmia and SCD risks compared to ARVC from other gene variants.^51^^,^^67^ Recent research highlights genotype-specific risk factors, influencing the application of ARVC risk calculators across patient subgroups.^68^

Although no recent studies have specifically focused on these syndromes, recommendations are extrapolated from research on nonsyndromic ARVC. According to the ESC guidelines for cardiomyopathies, ICD implantation is recommended for secondary prevention in ARVC patients who have survived cardiac arrest or experienced ventricular arrhythmia with hemodynamic instability (Class I, Level of Evidence: A) and should be considered for those with hemodynamically tolerated ventricular tachycardia (Class IIa, Level of Evidence: B).

For primary prevention, high-risk features and the 2019 ARVC risk calculator guide individualized ICD decisions, especially for JUP variants, which show no major differences compared to PKP2 cases.^69^ In *DSP* variants, a gene-first risk stratification approach allows for tailored ventricular arrhythmia risk assessment, providing a more personalized approach to prevention.^70^

As arrhythmic risk in ARVC evolves over time, regular re-evaluation is essential. Upon identification of the specific cutaneous features, a cardiac evaluation and yearly follow-up with 12-lead resting ECG, 24-hour Holter monitoring, CMR or, if not applicable, 2D-echocardiography is required. Longitudinal studies are needed to define the optimal frequency of follow-up in Naxos disease and similar syndromes. Additionally, a dedicated risk model for these syndromes remains greatly needed and would be facilitated by developing a Registry for Cardiocutaneous Syndromes. Such a registry would be crucial, providing essential data on disease progression and treatment outcomes to improve patient care.

#### Lifestyle modification

The interplay between genotype and exercise in ARVC shows a dose-dependent relationship where more intense exercise increases disease risk and adverse outcomes in desmosomal variant carriers.^71^ Endurance sports are linked to earlier disease onset, pronounced structural abnormalities, higher risk of ventricular arrhythmias, and increased heart failure risk in ARVC patients.^72^, ^73^, ^74^

There is an exercise threshold beyond which disease phenotypes appear; thus, individuals with desmosomal variants may engage safely in low-to-moderate intensity exercise.^75^ Although specific guidelines for Naxos disease are unavailable, patients should avoid competitive sports and high-intensity exercise.

#### Management of noncardiac disease

Management of syndromic palmoplantar keratosis in Naxos disease focuses on symptom relief, as no specific cure exists. Treatments aim to soften and smooth thickened skin, improve function and appearance, and reduce discomfort. Methods include regular soaks and gentle scale removal. Topical keratolytics like urea, lactic acid, salicylic acid, and propylene glycol are used cautiously in children. Retinoids (eg, tazarotene, tretinoin) are reserved for severe cases due to side effects and offer temporary relief.^76^

#### Multidisciplinary team, education, and network organization leveraging artificial intelligence in managing Naxos disease and related syndromes

Managing Naxos disease and related cardiocutaneous syndromes necessitates a MDT team and guideline-based approach for accurate diagnosis, family screening, and ongoing care.^5^ Effective networks should overcome diagnostic delays, coordinate care across various health care levels, and ensure comprehensive patient support while addressing geographic and socioeconomic diversity.

AI is revolutionizing cardiac disease management^77^ by enhancing screening, streamlining referrals, and aiding nuanced diagnosis and tailored management. Successful AI implementation requires robust data sets and collaboration among clinical experts, biomedical engineers, and AI specialists to advance patient care for these syndromes.

### The unmet need: patients’ perspective

A cardiovascular disease diagnosis, especially 1 with sudden death as a first symptom, is shocking, particularly for young individuals and athletes. While doctors treat the disease symptoms and prognosis, patients often struggle with the psychosocial burden. The fear, lifestyle restrictions, and work limitations cause anxiety and stress, negatively impacting mental health. Early-stage psychosocial support in clinics is essential for maintaining mental health, productivity, and quality of life. Long-term comprehensive psychosocial support is crucial to relieve the mental and emotional unrest caused by Naxos disease and similar inherited cardiocutaneous syndromes.

## Conclusions

Naxos disease and related cardio-cutaneous syndromes are rare, globally distributed conditions caused by genetic variants affecting desmosomal proteins, leading to arrhythmogenic cardiomyopathy and skin abnormalities. The pathophysiology involves disrupted cellular adhesion and significant alterations in intracellular signaling pathways, contributing to complex cardiac and skin manifestations. Effective management requires early recognition, precise genetic identification, individualized treatment, and psychosocial support. Despite limited understanding of their epidemiology and clinical diversity, advancements in molecular technologies and targeted therapies highlight the need for an international registry to facilitate collaborative research, share best practices, and improve patient care and quality of life.

## Funding support and author disclosures

Dr Protonotarios holds consulting agreements with Tenaya Therapeutics and Avidity Biosciences. Dr Crisci has received a research grant by the CardioPath program from Federico II University of Naples, Italy. Dr Abrams is a consultant/scientific advisory board for Thryv Therapeutics, Rockets Pharmaceuticals, Solid Biosciences. Dr Anastasakis is on the advisory board of Pfizer, Genesis, Takeda, Sanofi, and BMS. Dr Mestroni has received grant support from Tenaya Therapeutics, Pfizer, AstraZeneca, Owkin, Greenstone Bioscience, and Bristol Myers Squibb; and is a member of the scientific advisory board of Tenaya Therapeutics. Dr Saffitz is consultant for Implicit Bioscience and Rocket Pharmaceuticals. Dr McKenna is a scientific advisor to Health in Code and to Tenaya Therapeutics. Dr Elliott holds consultancies in Biomarin, Pfizer, BMS, and the Patent: US Pat No 62/903,103: GENE THERAPY COMPOSITIONS AND TREATMENT OF RIGHT VENTRICULAR ARRYTHMOGENIC CARDIOMYOPATHY. Dr Kaski has consultancy/advisory board activities with Tenaya Therapeutics, Cytokinetics, Lexeo, Avidity Biosciences, and Accord Healthcare. All other authors have reported that they have no relationships relevant to the contents of this paper to disclose.
