# Supplementary material for: Naxos Disease and Related Cardio-Cutaneous Syndromes
Source: JACC Adv. 2025 Jan 10;4(2):101547. doi: 10.1016/j.jacadv.2024.101547 (PMC11773020; doi:10.1016/j.jacadv.2024.101547)
Supplement: Supplementary data [file mmc1.docx]

**Supplementary Table.** Naxos disease and related cardio-cutaneous syndromes. Publication, clinical and genetic features of all published cases up to 07/2024.

| ID | Author,  Year | Co** | Age  year | S | Gene | Variant | Z | CP | Symptoms, events | ECG | ECHO | CMR | FU, yrs | Outcome | CH | Cutaneous manifestation,  Onset | |
| --- | --- | --- | --- | --- | --- | --- | --- | --- | --- | --- | --- | --- | --- | --- | --- | --- | --- |
| 1 | Protonotarios, 2001^1^ | GR | 7 | M | *JUP* | c.2157del2 | Ho | ARVC | No | TWI V1-V3 | RV, progression° | NR | 16 | No  Events | NR | Infancy, PPK, WH | |
| 2 | Protonotarios, 2001^1^ | GR | 17 | M | *JUP* | c.2157del2 | Ho | ARVC | Syncope | TWI V1-V6 | RV progression° | NR | 11 | rVT,  HFD | NR | Infancy, PPK, WH | |
| 3 | Protonotarios, 2001^1^ | GR | 32 | F | *JUP* | c.2157del2 | Ho | ARVC | Syncope | TWI V1-V4 | RV, LV progression° | NR | 16 | rVT, ICDs | NR | Infancy, PPK, WH | |
| 4 | Protonotarios, 2001^1^ | GR | 29 | M | *JUP* | c.2157del2 | Ho | ARVC | Syncope | TWI V1-V4 | RV, LV progression° | NR | 15 | rVT, HF, ICDs | NR | Infancy, PPK, WH | |
| 5 | Protonotarios, 2001^1^ | GR | 17 | M | *JUP* | c.2157del2 | Ho | ARVC | Syncope | TWI V1-V3 | RV progression° | NR | 11 | rVT, HF, SCD | RV + | Infancy, PPK, WH | |
| 6 | Protonotarios, 2001^1^ | GR | 41 | F | *JUP* | c.2157del2 | Ho | ARVC | Syncope,  Dyspnoea | TWI V1-V5 | RV, LV progression° | NR | 12 | NSVT,  HF, SCD | RV+,  LV+ | Infancy, PPK, WH | |
| 7 | Protonotarios, 2001^1^ | GR | 36 | F | *JUP* | c.2157del2 | Ho | ARVC | No | TWI V1 | RV | NR | 15 | No Events | NR | Infancy, PPK, WH | |
| 8 | Protonotarios, 2001^1^ | GR | 43 | M | *JUP* | c.2157del2 | Ho | ARVC | Palp | NR | RV, LV progression° | NR | 13 | rVT, HF | NR | Infancy, PPK, WH | |
| 9 | Protonotarios, 2001^1^ | GR | 35 | M | *JUP* | c.2157del2 | Ho | ARVC | Chest Pain | TWI V1-V4 | RV, LV progression° | NR | 15 | SCD | NR | Infancy, PPK, WH | |
| 10 | Protonotarios, 2001^1^ | GR | 32 | F | *JUP* | c.2157del2 | Ho | ARVC | No | TWI V1-V3 | RV progression° | NR | 16 | No Events | NR | Infancy, PPK, WH | |
| 11 | Protonotarios, 2001^1^ | GR | 74 | M | *JUP* | c.2157del2 | Ho | ARVC | Syncope | TWI V1-V5 | RV progression° | NR | 7 | No Events | NR | Infancy, PPK, WH | |
| 12 | Protonotarios, 2001^1^ | GR | 61 | F | *JUP* | c.2157del2 | Ho | ARVC | Syncope | TWI V1-V4 | RV progression° | NR | 16 | NSTV | NR | Infancy, PPK, WH | |
| 13 | Protonotarios, 2001^1^ | GR | 56 | F | *JUP* | c.2157del2 | Ho | ARVC | Syncope | TWI V1-V3 | RV progression° | NR | 15 | VES | NR | Infancy, PPK, WH | |
| 14 | Protonotarios, 2001^1^ | GR | 49 | F | *JUP* | c.2157del2 | Ho | ARVC | Syncope | TWI V1-V6 | RV progression° | NR | 12 | Recurrent VT | NR | Infancy, PPK, WH | |
| 15 | Protonotarios, 2001^1^ | GR | 40 | M | *JUP* | c.2157del2 | Ho | ARVC | Syncope | TWI V1-V5 | RV, LV progression° | NR | 15 | VES | NR | Infancy, PPK, WH | |
| 16 | Protonotarios, 2001^1^ | GR | 60 | M | *JUP* | c.2157del2 | Ho | ARVC | No | TWI V1-V4 | RV progression° | NR | 12 | No Events | NR | NR Infancy, PPK, WH | |
| 17 | Protonotarios, 2001^1^ | GR | 26 | M | *JUP* | c.2157del2 | Ho | ARVC | Syncope | TWI V1-V6 | RV, LV ° | NR | 1 | SCD | NR | Infancy, PPK, WH | |
| 18 | Protonotarios, 2001^1^ | GR | 19 | M | *JUP* | c.2157del2 | Ho | ARVC | Palp | TWI V1-V6 | RV, LV progression° | NR | 14 | rVT, HFD | RV + LV+ | Infancy, PPK, WH | |
| 19 | Protonotarios, 2001^1^ | GR | 16 | F | *JUP* | c.2157del2 | Ho | ARVC | Syncope | LQRSV  TWI inferior leads | RV, LV° | NR | 1 | HF, SCD | NR | Infancy, PPK, WH | |
| 20 | Protonotarios, 2001^1^ | GR | 47 | M | *JUP* | c.2157del2 | Ho | No | No | TWI V1-V4 | RV alterations° | NR | 8 | No Events | NR | Infancy, PPK, WH | |
| 21 | Protonotarios, 2001^1^ | GR | 45 | F | *JUP* | c.2157del2 | Ho | No | No | TWI V1-V3 | RV alterations° | NR | 4 | No Events | NR | Infancy, PPK, WH | |
| 22 | Protonotarios, 2001^1^ | GR | 15 | F | *JUP* | c.2157del2 | Ho | ARVC | VES, Syncope | TWI V1-V2 | RV, LV° | NR | 5 | SCD | RV+  LV+ | Infancy, PPK, WH | |
| 23 | Protonotarios, 2001^1^ | GR | 17 | F | *JUP* | c.2157del2 | Ho | No | Syncope | TWI V1-V3 | RV progression° | NR | 4 Years | ICD | NR | Infancy, PPK, WH | |
| 24 | Protonotarios, 2001^1^ | GR | 58 | F | *JUP* | c.2157del2 | Ho | No | No | TWI V1-V6 | RV alterations° | NR | 2 Years | No Events | NR | Infancy, PPK, WH | |
| 25 | Protonotarios, 2001^1^ | GR | 13 | M | *JUP* | c.2157del2 | Ho | No | Syncope | TWI V1-V4 | RV alterations° | NR | 2 Years | No Events | NR | Infancy, PPK, WH | |
| 26 | Protonotarios, 2001^1^ | GR | 5 | F | *JUP* | c.2157del2 | Ho | No | No | TWI V1-V2 | RV alterations° | NR | 2 Years | No Events | NR | Infancy, PPK, WH | |
| 27 | Protonotarios, 2001^1^ | GR | 39 | F | *JUP* | c.2157del2 | Ho | No | No | TWI V1-V2 | RV alterations° | NR | 2 Years | No Events | NR | Infancy, PPK, WH | |
| 28 | Protonotarios, 2001^1^ | GR | 1 | M | *JUP* | c.2157del2 | Ho | No | No | TWI V1-V2 | RV alterations° | NR | 3 Years | No Events | NR | Infancy, PPK, WH | |
| 29 | Narin, 2003^2^ | TR | 13 | M | *JUP* | c.2157del2 | Ho | ARVC | Syncope | TWI V1-V6 | RV | NR | NR | VT | NR | Infancy, PPK, WH | |
| 30 | Lazaros, 2009^3^ | GR | 43 | M | *JUP* | c.2157del2 | Ho | ARVC | Syncope | TWI V1-V4 | RV, LV | RV, LV | NR | VT,  ICD | NR | Infancy, PPK, WH | |
| 31 | Erken, 2011^4^ | TR | 34 | M | *JUP* | c.794G>A | Ho | ARVC | Syncope,  Dyspnoea | TWI precordial | RV, LV | RV, LV | 3 m | VT, | RV (+) | Infancy, PPK, WH, WH | |
| 32 | Erken, 2001^4^ | TR | 46 | M | *JUP* | c.794G>A | Ho | ARVC | HF | TWI V1-V3 | RV | NR | NR | ICD | RV (+)  EMB | PPK, Alopecia |  |
| 33 | Baykan, 2015^5^ | TR | 13 | M | *JUP* | c.2157del2 | Ho | ARVC | Chest Pain, Sync | TWI | RV, LV | NR | 5 | rVT, ICD | NR | Infancy, PPK, WH |  |
| 34 | Baykan, 2015^5^ | TR | 6 | M | *JUP* | c.2157del2 | Ho | No | Chest Pain | NSVT | Normal | NR | 5 | No Events | NR | Infancy, PPK, WH |  |
| 35 | Baykan, 2015^5^ | TR | 1.5 | F | *JUP* | NR | Ho | No | NR | NR | NR | NR | NR | NR | NR | Toddler, PPK, WH,  Dystrophic Nails |  |
| 36 | Baykan, 2015^5^ | TR | 3 | M | *JUP* | c.2157del2 | Ho | DCM | Dyspnoea |  | LV, RV | NR | 0 | SCD | NR | Toddler, PPK, WH |  |
| 37 | Boente, 2016^6^ | AR | 17 | M | *JUP* | c.71C>A | Ho | ARVC | NR | NR | RV, LV | NR | NR | NR | NR | PPK, WH,  Oligodontia  Nail Dystrophy |  |
| 38 | Sonsoz, 2021^7^ | TR | 19 | F | *JUP* | c.901_903delGAG | Ho | ARVC | NR | TWI V1-V6 | RV, LV | LV, RV (LVEF 21%). LV, RV LGE | NR | ICD | NR | PPK, WH,  Sparse Eyebrows |  |
| 39 | Sonsoz, 2021^7^ | TR | 15 | M | *JUP* | c.901_903delGAG | Ho | No | NR | NR | Normal | Normal | NR | NR | NR | PPK, WH,  Nail Dysplasia  Sparse Eyebrows |  |
| 40 | Te Riele ASJM, 2021^8^ | GR | 1.6 | M | *JUP* | c.2157del2 | Ho | No | no | Normal | Normal | Not done | 5 years | 3 VES in Holter | No | Infancy, PPK, dorsal eczematous lesions, WH |  |
| 41 | Alcalai, 2003^9^ | IL | 16 | F | *DSP* | c.7402G>C | Ho | ARVC | Syncope | TWI V1-V3, VT | RV | NR | NR | ICD | NR | PPK, WH |  |
| 42 | Uzumcu, 2006^10^ | TR | 3 | M | *DSP* | c.3799C>T | Ho | DCM | NR | NSVT | LV, RV (LVEF <25%) | NR | NR | HFD | NR | Infancy, PPK, WH |  |
| 43 | Norgett, 2006^11^ | UK | 3 | M | *DSP* | c.1817_1846dup | He | DCM | NR | NSVT | LV | NR | 4 Years | ICD, SCD | NR | Toddler, PPK, WH,  Oligodontia |  |
| 44 | Prompona, 2007^12^ | GER | 12 | M | *DSP* | Deletion in Exon 23 | Ho | DCM | NR | TWI V2-V6, | LV, RV (LVEF < 20%), | LV, RV (LVEF 24%).  LV LGE | NR | Heart Transplantation | RV, LV + | Infancy, PPK, WH |  |
| 45 | Alonso-Orgaz, 2007^13^ | ES | 43 | M | *DSP* | c.2447A>C | He  2 | ARVC | Palp. Syncope | TWI  II,III,aVF  V1-V6 | NR | NR | NR | NR | NR | Infancy, PPK,WH |  |
| 46 | Tanaka, 2009^14^ | BR | 5 | F | *DSP* | c.2516del; c.3971del | He  2 | DCM | Dyspnoea | Frequent PVCs | RV, LV | NR | NR | NR | NR | Infancy, PPK, WH  Thick nails, Alopecia |  |
| 47 | Mahoney, 2010^15^ | FI | 14 | F | *DSP* | c.6310del; c.7964C>A | He2 | DCM | Asymptomatic | NR | NR | NR | NR | SCD (sleep) | LV, RV  + | Adolescence, PPK, WH,  Enamel Defects  Dystrophy and Thickening of the Toenails |  |
| 48 | Christensen, 2010^16^ | DK | 35 | M | *DSP* | c.6166G>C | Ho | Biventricular ARVC | Palp. Dyspnoea | NR | NR | NR | NR | NR | NR | PPK, Curly hair |  |
| 49 | Chalabreysse, 2011^17^ | FR | 17 | M | *DSP* | c.1790C>T | He | DCM | Syncope (Exercise) | IRBBB, NSVT | Dilated LV (LVEF 20%) | NR | 4 Years | LV progression (LVEF 20%), Heart Transplantation | LV, RV  + | PPK, WH,  Oligodontia |  |
| 50 | Chalabreysse, 2011^17^ | FR | NR | M | *DSP* | c.1790C>T | He | No | Syncope (Exercise) | IVCD | LV Dilation (LVEF 55%) | NR | NR | NR | NR | PPK, WH, Oligodontia |  |
| 51 | Williams, 2011^18^ | GER | 6 | M | *DSP* | c.5208del | Ho | DCM | NR | NR | LV (LVEF 11%) | NR | 2 Years | HFD | NR | Toddler, PPK, WH |  |
| 52 | Williams, 2011^18^ | GER | 5 | M | *DSP* | c.5208delAG | Ho | DCM | NR | NR | LV  (LVEF 26%) | LV (LVEF 26%)  LV LGE | 5 Years | Heart Transplantation | RV+  LV+ | Toddler, PPK |  |
| 53 | Krishnamurthy, 2011^19^ | IN | 11 | F | *DSP* | c.3901C>T | Ho | DCM | Palp,  Dyspnoea | Frequent PVCs | RV, LV  (LVEF 30%) | NR | NR | NR | NR | PPK, WH |  |
| 54 | Boulé, 2012^20^ | FR | 29 | M | *DSP* | c.1691C>T | He | ARVC | Asymptomatic | TWI II,  III, aVF, V4-V6, Freq VES | LV (LVEF 35%). RV Aneurysm | RV, LV LGE +_ | NR | ICD | NR | Infancy, PPK, WH,  Oligodontia  Leukonychia |  |
| 55 | Boulé, 2012^20^ | FR | 10 | M | *DSP* | c.1691C>T | He | ARVC | Chest Pain | PVCs, Epsilon | RV | RV LGE | NR | NR | NR | PPK, WH, Oligodontia  Brittle Nails |  |
| 56 | Keller, 2012^21^ | CH | 7 | M | *DSP* | c.1748T>C | He | DCM | NR | NR | RV, LV  (LVEF 19%). | RV, LVLGE | 7 | ICDs | NR | Toddler, PPK, WH  Dystrophic Nails  Sparse Eyebrows |  |
| 57 | Keller, 2012^21^ | CH | 13 | F | *DSP* | c.1691C>T | He | DCM | Syncope | IVCD Epsilon PVCs | RV, LV | NR | 9 | ICD | NR | PPK, WH,  Oligodontia, Leukonychia |  |
| 58 | Rasmussen, 2013^22^ | DK | 8 | M | *DSP* | c.7780del | Ho | DCM | HF | NR | LV, RV  (LVEF 15%) | NR | 5 | Heart Transplantation | RV+  LV+ | Childhood, PPK, WH |  |
| 59 | Yesudian, 2014^23^ | UK | 2 | F | *DSP* | c.7567del; c.6577G>A | HE2 | DCM | NR | LV | NR | NR | NR | NR | NR | Infancy, PPK,  Alopecia |  |
| 60 | Baykan, 2015^24^ | TR | 6 | M | *DSP* | NR | NR | DCM | Dyspnoea | 1st AV Block, TWI V1-V3 | RV, LV  (LVEF 27%) | NR | 2 | HFD | NR | Childhood, PPK, WH |  |
| 61 | Molho-Pessach, 2015^25^ | IL | 4 | M | *DSP* | c.3924del | Ho | DCM | Dyspnoea | Frequent PVCs | LV | RV, LV, LVLGE | NR | ICD | NR | Infancy, PPK, WH |  |
| 62 | Molho-Pessach, 2015^25^ | IL | 59 | F | *DSP* | c.7111C>A | Ho | ARVC | Chest Pain, Syncope | NR | RV, LV | NR | NR | ICD | NR | Infancy  PPK, WH,  Onychogryphosis, Sparse Eyebrows |  |
| 63 | Molho-Pessach, 2015^25^ | IL | 21 | F | *DSP* | c.7111C>A | Ho | DCM | Chest Pain, Syncope | Frequent PVCs, Couples and Triplets | LV, RV | NR | NR | SCD | NR | Infancy, PPK, WH,  Sparse Eyebrows |  |
| 64 | Pigors, 2015^26^ | RO | 5 | M | *DSP* | c.7566_7567delAAinsC; c.7756C>T | He2 | No | No | No | No | NR | NR | NR | NR | Toddler, PPK,  Hypotrichosis |  |
| 65 | Pigors, 2015^26^ | GER | 14 | M | *DSP* | c.1067C>A; c.2131_2132del | He2 | DCM | HF | NR | NR | NR | NR | Heart Transplantation | NR | Infancy, PPK, WH |  |
| 66 | Pigors, 2015^25^ | GER | 10 | F | *DSP* | c.1067C>A; c.2131_2132del | He2 | DCM | NR | NR | NR | NR | NR | NR | NR | Infancy, PPK,  Alopecia |  |
| 67 | Bitar, 2016^28^ | USA | 17 | M | *DSP* | c.1865T>C | He2 | DCM | No | NR | LV (LVEF 25%) | NR | NR | NR | NR | Infancy, PPK, WH  Oligodontia  Leukonychia |  |
| 68 | Finsterer, 2016^29^ | AT | 43 | F | *DSP* | c.1678A>T | He | DCM | NR | NR | LV | NR | NR | NR | NR | Adolescence, PPK, WH,  Oligodontia |  |
| 69 | Finsterer, 2016^29^ | AT | NR | M | *DSP* | c.1678A>T | He | DCM | Recurrent Syncope | NR | NR | NR | NR | Cardiac Death | RV+ | Dental abnormalities |  |
| 70 | Ramoglu, 2017^30^ | TR | 5 | F | *DSP* | c.4650_4651del | Ho | DCM | Dyspnoea | NR | LV, RV,  (LVEF 25%) | NR | 2 | Heart Transplantation | NR | PPK, WH |  |
| 71 | Erolu, 2018^31^ | TR | 4 | M | *DSP* | c.3564T>A; c.4395T>A | He2 | DCM | Palp | IVCD Epsilon Waves | RV, LV (LVEF 45%) | RV, LVLGE | NR | VT, ICD | NR | PPK,WH,  Sparse Eyebrows |  |
| 72 | Akdogan, 2018^32^ | TR | 8 | F | *DSP* | c.4650_4651del | Ho | DCM | NR | Polymorphic PVC | LV (LVEF 25%) | NR | NR | NR | NR | Toddler, PPK, WH  Leukonychia |  |
| 73 | Akdogan, 2018^32^ | TR | 5 | F | *DSP* | c.4650_4651del | Ho | DCM | NR | NR | NR | NR | NR | NR | NR | Toddler, PPK,WH |  |
| 74 | Ou, 2022^33^ | FR | 13 | F | *DSP* | c.1782C>G | He | ARVC | NR | Frequent PVCs | NR | RV (RVEF 32%) | NR | NR | NR | Infancy, PPK, WH  Oligodontia  Fragile Nails |  |
| 75 | Zhao, 2023^34^ | CN | 7 | F | *DSP* | c.4597C>T | Ho | DCM | Dyspnoea | IVCD Frequent PVCs | RV, LV (LVEF 25%) | RV, LV | NR | NR | NR | Toddler, PPK, WH  Oligodontia  Leukonychia |  |
| 76 | Kincaid, 2023^35^ | CA | 37 | M | *DSP* | c.2652_2653del | He | NR | Chest Pain, Palp | TWI, NSTV (exercise) | LV | Fibrofatty Infiltration | NR | ICD | NR | PPK, WH |  |
| 77 | Kincaid, 2023^35^ | CA | 34 | M | *DSP* | c.2652_2653del | He | NR | NR | NR | NR | No | NR | NR | NR | PPK, WH |  |
| 78 | Kincaid, 2023^35^ | CA | 44 | F | *DSP* | c.2652_2653del | He | NR | NR | NR | NR | NR | NR | NR | NR | PPK, WH |  |
| 79 | Kincaid, 2023^35^ | CA | 1 | F | *DSP* | c.2652_2653del | He | NR | NR | NR | NR | NR | NR | NR | NR | curly hair |  |
| 80 | Kincaid, 2023^35^ | CA | 3 | F | *DSP* | c.2652_2653del | He | NR | NR | NR | NR | NR | NR | NR | NR | Focal Hyperkeratosis |  |
| 81 | Kincaid, 2023^35^ | CA | 4 | M | *DSP* | c.2652_2653del | He | NR | NR | NR | NR | NR | NR | NR | NR | Focal Hyperkeratosis |  |
| 82 | Kincaid, 2023^35^ | CA | 2 | F | *DSP* | c.2652_2653del | He | NR | NR | NR | NR | NR | NR | NR | NR | Focal Hyperkeratosis |  |
| 83 | Kincaid, 2023^35^ | CA | Months | F | *DSP* | c.2652_2653del | He | NR | NR | NR | NR | NR | NR | NR | NR | NR |  |
| 84 | Norget 2000^37^  Carvajal-Huerta, 1998^36^  _Kaplan 2004_ ^59^ | EC | 10 | F | *DSP* | c.7901del | Ho | DCM | NR | LQRSV,  TWI V1-V4,  III, aVF,  PVCs | LV | NR | 1 | HFD | RV, LV, aneurysms, fibrosis | Infancy, PPK, WH |  |
| 85 | Norget 2000^37^  Carvajal-Huerta, 1998^36^ | EC | 12 | F | *DSP** | c.7901del | Ho | DCM | NR | Abnormal | LV | NR | 5 Years | HFD | NR | Infancy, PPK, WH |  |
| 86 | Norget 2000^37^  Carvajal-Huerta, 1998^36^ | EC | 14 | F | *DSP** | c.7901del | Ho | DCM | NR | Abnormal, Frequent PVCs and NSVT | LV | NR | NR | NR | NR | Infancy, PPK, WH |  |
| 87 | Norget 2000^37^  Carvajal-Huerta, 1998^36^ | EC | 8 | F | *DSP** | c.7901del | Ho | DCM | NR | Abnormal, Frequent PVCs and Couplets | LV | NR | NR | NR | NR | Infancy, PPK, WH |  |
| 88 | Norget 2000^37^  Carvajal-Huerta, 1998^36^ | EC | 10 | M | *DSP** | c.7901del | Ho | No | NR | Abnormal, Frequent PVCs | Normal | NR | NR | NR | NR | Infancy, PPK, WH |  |
| 89 | Carvajal-Huerta, 1998^36, 37^ | EC | 13 | M | *DSP** | c.7901del | Ho | DCM | NR | Abnormal | LV | NR | 4 Years | HFD | NR | Infancy, PPK, WH |  |
| 90 | Carvajal-Huerta, 1998^36^ | EC | 32 | F | NR | NR | NR | No | NR | Abnormal | Normal | NR | NR | NR | NR | Infancy, PPK, WH |  |
| 91 | Carvajal-Huerta, 1998^36^ | EC | 34 | F | *NR* | NR | NR | No | NR | Abnormal | Normal | NR | NR | NR | NR | Infancy, PPK, WH |  |
| 92 | Carvajal-Huerta, 1998^36^ | EC | 11 | F | *NR* | NR | NR | DCM | NR | Abnormal | LV | NR | 1 Year | Advanced HF-Related Death | NR | Infancy, PPK, WH |  |
| 93 | Carvajal-Huerta, 1998^36^ | EC | 12 | F | *NR* | NR | NR | DCM | NR | Abnormal | LV | NR | NR | Advanced HF-Related Death | NR | Infancy, PPK, WH |  |
| 94 | Carvajal-Huerta, 1998^36^ | EC | 14 | F | *NR* | NR | NR | DCM | NR | Abnormal, Frequent PVCs and Couplets | LV | NR | NR | NR | NR | Infancy, PPK, WH |  |
| 95 | Carvajal-Huerta, 1998^36^ | EC | 17 | F | *NR* | NR | NR | DCM | NR | Abnormal, Frequent PVCs and Couplets | LV | NR | NR | NR | NR | Infancy, PPK, WH |  |
| 96 | Bukhari, 2003^38^ | SA | 2 | F | NR | NR | NR | No | NR | NR | Normal | NR | NR | NR | NR | Toddler, PPK, WH,  Thin Nails  Sparse Eyebrows |  |
| 97 | Sajeev, 2006^39^ | IN | 55 | F | NR | NR | NR | ARVC | Palp, Syncope | TWI V1-V4 | RV Aneurysms | NR | NR | Sustained VT | NR | PPK, WH |  |
| 98 | Adhisivam, 2006^40^ | IN | 2 | F | NR | NR | NR | DCM | HF | Normal | RV, LV Dysfunction (LVEF 40%) | NR | 0 | HFD | NR | Infancy, PPK, WH |  |
| 99 | Kilic, 2007^41^ | TR | 17 | M | NR | NR | NR | ARVC | Dyspnoea, Palp | TWI V1-V3, Epsilon Waves | RV, LV | RV, LV | NR | ICD | NR | Adolescent, PPK, WH |  |
| 100 | Kilic, 2007^41^ | TR | NR | M | NR | NR | NR | ARVC | NR | Epsilon Waves | NR | NR | NR | NR | NR | No |  |
| 101 | Kilic, 2007^41^ | TR | NR | M | NR | NR | NR | ARVC | NR | NR | RV | RV | NR | NR | NR | PPK |  |
| 102 | Rai, 2008^42^ | IN | 7 | M | NR | NR | NR | No | NR | TWI V1-V2 | RV and RA Dilation | NR | NR | NR | NR | Infancy.PPK,WH |  |
| 103 | Kolar, 2008^43^ | UK | 16 | F | NR | NR | NR | DCM | NR | NR | NR | NR | NR | SCD | RV, LV Fibrosis and Myocyte Loss. | Adolescence, PPK, WH |  |
| 104 | Tanaka, 2009^14^ | BR | 10 | M | *PKP1* | c.2014C>T | Ho | NR | NR | NR | NR | NR | NR | NR | NR | Toddler, PPK, WH  Thickening of the Fingernails and Toenails |  |
| 105 | Simpson, 2009^44^ | UK | 29 | M | *DSC2* | c.1841del | Ho | ARVC | Syncope, Resuscitated SCD | IVCD  TWI V2, III, aVF, Frequent PVCs | LV (LVEF 45%), RV Dilation and Dysfunction | LV (LVEF 50%) RV (RVEF 32%) RV, LV LGE | NR | ICD | NR | PPK WH |  |
| 106 | Simpson, 2009^44^ | UK | 25 | F | *DSC2* | c.1841del | Ho | ARVC | NR | IVCD Biphasic TW in V2, Frequent PVCs | Normal | RV+/- | NR | NR | NR | PPK, WH |  |
| 107 | Koumantaki, 2010^45^ | GR | 54 | F | NR | NR | NR | NR | HF | NR | NR | NR | NR | ICD | NR | PPK, WH |  |
| 108 | Chalabreysse, 2011^17^ | FR | 15 | M | NR | NR | NR | No | Chest Pain | ST Elevation in V3-V4, PVCs | No | NR | NR | NR | NR | PPK, WH  Oligodontia |  |
| 109 | Noin, 2012^46^ | ES | 14 | M | NR | NR | NR | ARVC | AbortedSCD (Exercise) | IVCD | RV, Aneurysm | NR | 0 | ICD | NR | Infancy, PPK, WH  Yes |  |
| 110 | Saravanan, 2012^47^ | IN | 21 | M | NR | NR | NR | ARVC | VT | Epsilon Wave | RV | RV, RVLGE, | NR | VT | NR | PPK, WH |  |
| 111 | Barber, 2012^48^ | UK | 14 | F | NR | NR | NR | DCM | Asymptomatic | NR | NR | NR | NR | SCD (Exertion) | LV Dilation LV Patchy Fibrosis | Adolescence, PPK, WH,  White thickened nails  Sparse Eyebrows |  |
| 112 | Nehme, 2012^49^ | FR | 57 | M | NR | NR | NR | DCM | Dyspnoea | 1st AV Block | LV (LVEF 30%) and LVNC | NR | NR | NR | NR | Childhood, PPK, WH |  |
| 113 | Gultekin, 2013^50^ | TR | 54 | F | NR | NR | NR | ARVC | HF, Syncope | TWI V1-V3 Epsilon Waves. Polymorphic NSVT | RV (RVEF 14%). (LVEF 60%) | RV | NR | No Events | NR | PPK, WH  Alopecia |  |
| 114 | Salam, 2013^51^ | IN | 11 | F | NR | NR | NR | DCM | Syncope | CRBBB, LPFB | LV (LVEF 45%) | No LGE | 2 | Refractory Sustained VT, Progressive HF | NR | PPK, WH |  |
| 115 | Kaya, 2013^52^ | TR | 23 | F | NR | NR | NR | ARVC | Dyspnoea | LQRSV, TWI V2-V6 | RV, LV  (LVEF 23%) | RV, LV, LVLGE | NR | NR | NR | Childhood, PPK, WH |  |
| 116 | Yildiz, 2013^53^ | TR | 9 | F | NR | NR | NR | DCM | Syncope, Palp | NR | LV | NR | NR | VT | NR | Infancy, PPK, WH |  |
| 117 | Baykan, 2015^54^ | TR | 9 | F | NR | NR | NR | DCM | HF Palp, Syncope | NR | RV, LV | NR | 1 | SCD | NR | Childhood, PPK, WH |  |
| 118 | Islam, 2016^55^ | BD | 10 | M | NR | NR | NR | ARVC | Palp, Chest Pain, Progressive Dyspnoea | IRBBB, Epsilon Waves | RV Aneurysms | NR | 9m | VT, SCD | NR | PPK, WH |  |
| 119 | Dutta, 2016^56^ | IN | 4 | F | NR | NR | NR | ARVC | Recurrent Syncope, Dyspnoea | Frequent PVC, IVCD | RV, LV (LVEF 48%) | NR | NR | NR | NR | PPK, WH |  |
| 120 | Mandal, 2022^57^ | NP | 9 | F | NR | NR | NR | DCM | Dyspnoea | LQRSV, TWI, Frequent PVCs | LV (LVEF 20%) and RV dysfunction | NR | NR | NR | NR | Childhood, PPK, WH |  |

*Abbreviations:* ARVC, arrhythmogenic right ventricular cardiomyopathy; AF, atrial fibrillation; AVB, atrioventricular block; CH, cardiac histology; Co: Country; CP, cardiac phenotype; CRBBB, complete right bundle branch block; CMR, cardiac magnetic resonance; DCM, dilated cardiomyopathy; ECHO, echocardiography; EMB, endomyocardial biopsy; F, female; FH, family history; FW, free wall; HF, heart failure; HFD, Heart failure related death; He, heterozygosity; He2, compound heterozygosity; Ho, homozygosity; GER, Germany; GR, Greece; ICD, implantable cardioverter-defibrillator; ICDs, appropriate ICD shock; IRBBB, incomplete right bundle branch block; IV, interventricular; IVCD, Intraventricular conduction delay; LGE, late gadolinium enhancement; LPFB, left posterior fascicular block; LQRSV, low QRS voltages; LV, left ventricle; LVEF, left ventricular ejection fraction; m, months; M, male; NDLVC, non-dilated left ventricular cardiomyopathy; NR, not reported; NSVT, non-sustained ventricular tachycardia; Palp, palpitations; PPK, Palmoplantar keratoderma; PVC, premature ventricular complex; RA, right atrium; RV, right ventricle; rVT, recurrent VT; S, Sex; SCD, sudden cardiac death; Symptoms; TWI, T-wave inversion; VES, frequent ventricular extrasystoles; VT, ventricular tachycardia; WH, wolly hair; Z, zygosity.

°RV abnormalities including hypokinetic, akinetic or dyskinetic areas, diastolic bulging and trabecular disarrangement. Structural progression was defined as progressive alteration in ventricular dimensions and/or wall motion abnormalities during serial echocardiographic evaluation.

**: Countries are reported by international codes

**References**

1. Protonotarios N, Tsatsopoulou A, Anastasakis A, et al. Genotype-phenotype assessment in autosomal recessive arrhythmogenic right ventricular cardiomyopathy (Naxos disease) caused by a deletion in plakoglobin. J Am Coll Cardiol. 2001;38:1477–1484
2. Narin N, Akcakus M, Gunes T, et al. Arrhythmogenic right ventricular cardiomyopathy (Naxos disease): report of a Turkish boy. *Pacing Clin Electrophysiol*. 2003;26:2326–2329.
3. Lazaros G, Anastasakis A, Tsiachris D, Dilaveris P, Protonotarios N, Stefanadis C. Naxos disease presenting with ventricular tachycardia and troponin elevation. *Heart Vessels*. 2009;24:63–65
4. Erken H, Yariz KO, Duman D, et al. Cardiomyopathy with alopecia and palmoplantar keratoderma (CAPK) is caused by a *JUP* mutation. *British Journal of Dermatology*. 2011;165:917–921
5. Baykan A, Olgar Ş, Argun M, et al. Different clinical presentations of Naxos disease and Carvajal syndrome: Case series from a single tertiary center and review of the literature. *Anatol J Cardiol*. 2015;15:404–408
6. Boente MDC, Nanda A, Baselaga PA, Kelsell DP, McGrath JA, South AP. Cardiomyopathy diagnosed in the eldest child harbouring p.S24X mutation in JUP. *Br J Dermatol*. 2016;175:644–646
7. Sonsöz MR, İli EG, Gezdirici A, Topel C, Kahveci G, Bornaun H. A Rare Cause of Syncope: Naxos Disease Caused by Novel Homozygous Deletion in the JUP Gene. *Circ Cardiovasc Imaging*. 2021;14:e013059
8. Te Riele ASJM, James CA, Calkins H, Tsatsopoulou A. Arrhythmogenic Right Ventricular Cardiomyopathy in Pediatric Patients: An Important but Underrecognized Clinical Entity. *Front Pediatr*. 2021;9:750916
9. Alcalai R, Metzger S, Rosenheck S, Meiner V, Chajek-Shaul T. A recessive mutation in desmoplakin causes arrhythmogenic right ventricular dysplasia, skin disorder, and woolly hair. *J Am Coll Cardiol*. 2003;42:319–327
10. Uzumcu A, Norgett EE, Dindar A, et al. Loss of desmoplakin isoform I causes early onset cardiomyopathy and heart failure in a Naxos-like syndrome. *J Med Genet*. 2006;43:e5
11. Norgett EE, Lucke TW, Bowers B, Munro CS, Leigh IM, Kelsell DP. Early death from cardiomyopathy in a family with autosomal dominant striate palmoplantar keratoderma and woolly hair associated with a novel insertion mutation in desmoplakin. *J Invest Dermatol*. 2006;126:1651–1654
12. Prompona M, Kozlik-Feldmann R, Mueller-Hoecker J, Reiser M, Huber A. Images in cardiovascular medicine. Magnetic resonance imaging characteristics in Carvajal syndrome (variant of Naxos disease). *Circulation*. 2007;116:e524-530
13. Alonso-Orgaz S, Zamorano-León JJ, Fernandez-Arquero M, et al. Case report of a Spanish patient with arrhythmogenic right ventricular cardiomyopathy and palmoplantar keratoderma without plakoglobin and desmoplakin gene modifications. *Int J Cardiol*. 2007;118:275–277
14. Tanaka A, Lai-Cheong JE, Café MEM, et al. Novel truncating mutations in PKP1 and DSP cause similar skin phenotypes in two Brazilian families. *Br J Dermatol*. 2009;160:692–697
15. Mahoney MG, Sadowski S, Brennan D, et al. Compound heterozygous desmoplakin mutations result in a phenotype with a combination of myocardial, skin, hair, and enamel abnormalities. *J Invest Dermatol*. 2010;130:968–978
16. Christensen AH, Benn M, Bundgaard H, Tybjaerg-Hansen A, Haunso S, Svendsen JH. Wide spectrum of desmosomal mutations in Danish patients with arrhythmogenic right ventricular cardiomyopathy. *J Med Genet*. 2010;47:736–744
17. Chalabreysse L, Senni F, Bruyère P, et al. A new hypo/oligodontia syndrome: Carvajal/Naxos syndrome secondary to desmoplakin-dominant mutations. *J Dent Res*. 2011;90:58–64
18. Williams T, Machann W, Kühler L, et al. Novel desmoplakin mutation: juvenile biventricular cardiomyopathy with left ventricular non-compaction and acantholytic palmoplantar keratoderma. *Clin Res Cardiol*. 2011;100:1087–1093
19. Krishnamurthy S, Adhisivam B, Hamilton RM, Baskin B, Biswal N, Kumar M. Arrhythmogenic dilated cardiomyopathy due to a novel mutation in the desmoplakin gene. *Indian J Pediatr*. 2011;78:866–869
20. Boulé S, Fressart V, Laux D, et al. Expanding the phenotype associated with a desmoplakin dominant mutation: Carvajal/Naxos syndrome associated with leukonychia and oligodontia. *Int J Cardiol*. 2012;161:50–52
21. Keller DI, Stepowski D, Balmer C, et al. De novo heterozygous desmoplakin mutations leading to Naxos-Carvajal disease. *Swiss Med Wkly*. 2012;142:w13670
22. Rasmussen TB, Hansen J, Nissen PH, et al. Protein expression studies of desmoplakin mutations in cardiomyopathy patients reveal different molecular disease mechanisms. *Clin Genet*. 2013;84:20–30
23. Yesudian PD, Cabral RM, Ladusans E, et al. Novel compound heterozygous mutations in the *desmoplakin* gene cause hair shaft abnormalities and culminate in lethal cardiomyopathy. *Clin Exp Dermatol*. 2014;39:506–508
24. Baykan A, Olgar Ş, Argun M, et al. Different clinical presentations of Naxos disease and Carvajal syndrome: Case series from a single tertiary center and review of the literature. *Anatol J Cardiol*. 2015;15:404–408
25. Molho-Pessach V, Sheffer S, Siam R, et al. Two Novel Homozygous Desmoplakin Mutations in Carvajal Syndrome. *Pediatr Dermatol*. 2015;32:641–646
26. Pigors M, Schwieger-Briel A, Cosgarea R, et al. Desmoplakin mutations with palmoplantar keratoderma, woolly hair and cardiomyopathy. *Acta Derm Venereol*. 2015;95:337–340
27. Stöllberger C, Vujic I, Wollmann E, Freudenthaler J, Finsterer J. Carvajal syndrome with oligodontia, hypoacusis, recurrent infections, and noncompaction. *Int J Cardiol*. 2016;203:825–827
28. Bitar F, Najjar T, Hayashi R, et al. A novel heterozygous mutation in desmoplakin gene in a Lebanese patient with Carvajal syndrome and tooth agenesis. *J Eur Acad Dermatol Venereol*. 2016;30:e217–e219
29. Finsterer J, Stöllberger C, Wollmann E, Dertinger S, Laccone F. Autosomal dominant Carvajal plus syndrome due to the novel desmoplakin mutation c.1678A > T (p.Ile560Phe). *Mol Genet Metab Rep*. 2016;8:1–3
30. Ramoğlu MG, Uçar T, Ceylaner S, Atalay S, Tutar E. A novel mutation in the desmoplakin gene in two female siblings with a rare form of dilated cardiomyopathy: Carvajal syndrome. Anatol J Cardiol. 2017;18:435–436
31. Erolu E, Akalın F, Saylan Çevik B, Yücelten D. Arrhythmogenic right ventricular dysplasia, cutaneous manifestations and desmoplakin mutation: Carvajal syndrome. *Pediatr Int*. 2018;60:987–989
32. Akdogan N, Incel-Uysal P, Cavdarli B, Topcu V, Yalcin B. A case of Carvajal syndrome associated with cervical neuroblastoma in an 8-year-old girl. *Int J Dermatol*. 2019;58:611–613
33. Ou S, Cesarato N, Mauran P, et al. A new de novo heterozygous missense mutation in the desmoplakin gene, causing Naxos and Carvajal disease, associating oligodontia and nail fragility. *Clin Exp Dermatol*. 2022;47:1424–1426
34. Zhao X-J, Bai C-Y, Li X-Y, et al. A Novel Variant in the Desmoplakin Gene in One Case of the Rare Carvajal Syndrome with Dilated Cardiomyopathy: A Case Report and Literature Review. *Clin Cosmet Investig Dermatol*. 2023;16:2737–2748
35. Kincaid C, Horton L, Cheung B, Esse I, Gradus-Pizlo I, Mesinkovska NA. Desmoplakin mutation underlying autosomal dominant arrhythmogenic cardiomyopathy, palmoplantar keratoderma, and curly hair. *JAAD Case Rep*. 2023;36:56–59
36. Carvajal-Huerta L. Epidermolytic palmoplantar keratoderma with woolly hair and dilated cardiomyopathy. *J Am Acad Dermatol*. 1998;39:418–421
37. Norgett EE, Hatsell SJ, Carvajal-Huerta L, et al. Recessive mutation in desmoplakin disrupts desmoplakin-intermediate filament interactions and causes dilated cardiomyopathy, woolly hair and keratoderma. *Hum Mol Genet*. 2000;9:2761–2766
38. Bukhari I, Juma’a N. Naxos disease in Saudi Arabia. *J Eur Acad Dermatol Venereol*. 2004;18:614–616
39. Sajeev CG, Francis J, Sankar V, Vasudev B, Venugopal K. Images in cardiovascular medicine. Ventricular tachycardia: the spectrum continues to broaden: report of Naxos disease. *Circulation*. 2006;114:e60-61
40. Adhisivam B, Mahadevan S. Naxos disease. *Indian J Pediatr*. 2006;73:359–360
41. Kilic T, Babaoglu K, Aygün F, et al. Biventricular involvement in a Turkish boy with palmoplantar hyperkeratosis and curly hair, an unusual presentation of Naxos-Carvajal syndrome. *Int J Cardiol*. 2007;115:e122-125
42. Rai R, Ramachandran B, Sundaram VS, Rajendren G, Srinivas CR. Naxos disease: a rare occurrence of cardiomyopathy with woolly hair and palmoplantar keratoderma. *Indian J Dermatol Venereol Leprol*. 2008;74:50–52
43. Kolar AJO, Milroy CM, Day PF, Suvarna SK. Dilated cardiomyopathy and sudden death in a teenager with palmar-plantar keratosis (occult Carvajal syndrome). *J Forensic Leg Med*. 2008;15:185–188
44. Simpson MA, Mansour S, Ahnood D, et al. Homozygous mutation of desmocollin-2 in arrhythmogenic right ventricular cardiomyopathy with mild palmoplantar keratoderma and woolly hair. *Cardiology*. 2009;113:28–34
45. Koumantaki E, Gregoriou S, Kakrida M, Christofidou E, Katsambas A. What is your diagnosis? Diffuse nonepidermolytic palmoplantar keratoderma with woolly hair and cardiomyopathy (Naxos-Carvajal syndrome). *Cutis*. 2010;85:180, 189–190
46. Noain JAG, Golet AC, Calzada JN, Mellado AM, Duarte JM. Living after sudden death: A case report of Naxos disease. *Indian J Crit Care Med*. 2012;16:207–209
47. Saravanan RR, Amuthan V, Janarthanan RA, Balasubramanian S, Mohamed SN. A case of arrhythmogenic right ventricular cardiomyopathy-Naxos disease. *Indian Heart J*. 2012;64:84–87
48. Barber S, Day P, Judge M, Toole EO, Fayle S. Variant Carvajal syndrome with additional dental anomalies. *Int J Paediatr Dent*. 2012;22:390–396
49. Nehme N, El Malti R, Roux-Buisson N, Caignault J-R, Bouvagnet P. Evidence for genetic heterogeneity in Carvajal syndrome. *Cell Tissue Res*. 2012;348:261–264
50. Gultekin N, Kucukates E. An unusual form of Naxos disease and its improvement by adjuvant low-dose colchicine therapy. *Acta Cardiologica*. 2013;68:433–437
51. Salam AA, Remadevi KS, Kurup RP. Naxos disease and Carvajal variant. *Indian Pediatr*. 2013;50:596–598
52. Kaya H, Oylumlu M, Ertaş F, Cetinçakmak MG. Naxos disease: an unusual cause of cardiomyopathy. *Turk Kardiyol Dern Ars*. 2013;41:265
53. Yildiz H, Silay E, Coskuner I, et al. Anaesthesia in Naxos disease: first case report. *Bosn J Basic Med Sci*. 2013;13:63–65
54. Baykan A, Olgar Ş, Argun M, et al. Different clinical presentations of Naxos disease and Carvajal syndrome: Case series from a single tertiary center and review of the literature. *Anatol J Cardiol*. 2015;15:404–408
55. Islam AM, Rahman MT, Chowdhury AH. Cardiocutaneous syndrome (Naxos disease) in a Bangladeshi boy. *Cardiovasc Diagn Ther*. 2016;6:462–465
56. Dutta A, Ghosh SK, Majumder B, Majumdar R. Generalized woolly hair with diventricular arrythmogenic cardiomyopathy: a rare variant of Naxos disease. *Dermatology Online Journal*. 2016;22
57. Mandal KD, Shrestha PN, Ghimire A, Joshi P, Agrawal S, Shrestha P. Carvajal Syndrome- A Variant of Naxos Disease: A Case Report. *JNMA J Nepal Med Assoc*. 2022;60:187
